# How does team reflexivity affect new generation employee cooperative behavior in China? A cross-level moderated mediation model

**DOI:** 10.3389/fpsyg.2025.1365026

**Published:** 2025-03-27

**Authors:** Yuanrong Li, Hong Li, Ya Xiao

**Affiliations:** ^1^School of Economics and Management, University of Chinese Academy of Science, Beijing, China; ^2^Sino-Danish College, University of Chinese Academy of Sciences, Beijing, China

**Keywords:** team reflexivity, employee cooperative behavior, new generation employee, organizational trust, employee involvement climate, moderated mediation effect

## Abstract

**Introduction:**

Employee cooperative behavior is crucial for enterprises navigating uncertainty in the rapidly evolving digital era. Drawing on social information processing theory, this study examines the impact of team reflexivity on employee cooperative behavior, with a focus on the mediating role of organizational trust and the moderating role of employee involvement climate.

**Methods:**

A cross-level moderated mediation model was developed and tested using survey data from 412 employees across 84 project teams in China. Hierarchical linear modeling (HLM) was employed to analyze the proposed relationships.

**Results:**

The findings reveal that: (1) Team reflexivity significantly enhances employee cooperative behavior. (2) Organizational trust mediates the relationship between team reflexivity and employee cooperative behavior. (3) Employee involvement climate moderates the indirect effect, such that the mediation effect of organizational trust is stronger in teams with a higher level of employee involvement.

**Discussion:**

These results contribute to the understanding of how team reflexivity fosters cooperation among employees, highlighting the critical role of trust and the influence of organizational climate. The study provides theoretical and practical implications for fostering teamwork and trust in dynamic work environments.

## Introduction

With the advent of the digital era, enterprises are facing greater uncertainty due to the constantly rapid development of technology and the need to work more in the form of teams among employees (Teece et al., 1997; Maynard et al., 2018) and be more cooperative against threats from the external environment. However, the differences among team members tend to weaken employees’ trust and sense of belonging in the workplace (Chang et al., 2022), which is not conducive to cooperation among employees (Kramer et al., 1996), such as reducing the efficiency of the cooperation process. Additionally, for China, an emerging economy, the values and behaviors of employees in the organization vary significantly among generations (Ng et al., 2010; Huang et al., 2018), due to the rapid economic development, especially after the reform and opening up beginning in the 1980s. The new generation of employees in the post-1980s (Zhu et al., 2015; Ren et al., 2018), compared with the traditional and conservative employees before the reform and opening up, were more eager to realize their personal values, to be respected (Hou et al., 2018), and to pursue autonomy (Chen and Zhou, 2018) in their work while they are also more emotional, self-centered, lack self-control, and have a relatively insufficient sense of teamwork (Hou et al., 2014; Fang et al., 2019), which is not conducive to cooperative behaviors. Among these employees, those born in the post-1990s are becoming the majority of the Chinese workforce and demonstrate more individualism and self-centered characteristics, and have weaker team spirit and willingness to cooperate (Gursoy et al., 2008; Cameron et al., 2013; Kim et al., 2018). In addition, the empirical evidence shows that the impact of the one-child policy further poses a greater threat to teamwork in the workplace (Goh and Lee, 2018). At this point, teams in Chinese enterprises need not only to contend with external risks but also enhance internal employees’ willingness to cooperate, which can be achieved through effective team interaction (Byrne, 1993; Adler and Gundersen, 2001), so that it provides a possible approach to facilitate better integration and increased collaboration among the new generation of employees born in the 1990s. Therefore, how to adjust the management processes and measures to improve employee cooperative behavior has become a common concern of both academics and practitioners.

Existing literature shows that scholars have carried out a lot of research on the aspects of individual factors (such as individual characteristics, motivation, and expectation) and contextual factors (such as team culture, team climate, leadership, and team diversity) about employee cooperative behavior (Barrick and Mount, 1991; Chatman and Barsade, 1995; Gilbert et al., 2012; Lin et al., 2013; Liang et al., 2015; Anvuur and Kumaraswamy, 2016; Mulyani et al., 2020; Chen et al., 2022; Lin et al., 2022). However, in the organizational context, in addition to variables such as team culture and leadership, internal team interaction may also exert a systematic environmental impact on employee cooperative behavior (Salas et al., 2015). Internal team interaction is an important team process for teams and organizations to achieve their goals. It advocates creating effective communication and exchange among employees within the team to promote their ability and willingness to cooperate, to exert a systematic environmental impact on employee cooperative behavior (Ayoko, 2016; van Gerwen et al., 2018b). Some scholars have found that the implementation of internal team training can promote the formation of common norms among employees and increase their skills to help others, thus improving employee cooperative behavior (van Gerwen et al., 2018b). It is found that scholars pay more attention to how team training affects employee cooperative behavior aimed at improving organizational performance and the impact mechanism between the team training and employee cooperative behavior (Lambooij et al., 2007; van Gerwen et al., 2018b,a; Arumsari et al., 2023). Different from team training focusing on inculcating information and sense of teamwork into employees’ minds and making employees training receivers (Amirtharaj et al., 2011; van Gerwen et al., 2018b), team reflexivity pays more attention to the involvement of employees (Wang et al., 2021), which allows information to be actively captured by employees and common norms to be effectively established by incorporating employees into the cyclic process of reflection, planning, and action/adaptation (West, 1996; Konradt et al., 2016). Besides, the self-centered tendency of the new generation employees often weakens cooperative behavior which has an altruistic nature (Hou et al., 2014; Fang et al., 2019; Fu et al., 2019). Therefore, this study infers that team reflexivity will also have an impact on employee (especially new generation employees) cooperative behavior because it can counter the weakening effect by forming accurately shared understandings of tasks, goals, roles, and other aspects in the team via jointly discussing new problems and solutions in the team and implement them into action (West et al., 1997; Edmondson, 2002); and by clarifying collective goals and interests, improving the acceptance of collective interests and values through the combination of self-interests and collective interests, thus correspondingly strengthening the cooperation in a targeted way (Swift and West, 1998; Mohammed et al., 2010). Unfortunately, the mechanism between these two aspects remains unclear. Based on the above, this study focuses on the possible impact of team reflexivity on new generation employee cooperative behavior, as well as the mechanism and boundary conditions in between.

Previous research on the relationship between team interaction and cooperative behavior is mostly based on the social exchange theory (Lambooij et al., 2007; Blau, 2017; van Gerwen et al., 2018b). In order to further identify how team reflexivity affects employee cooperative behavior, this paper studies the internal mechanism of such impact based on social information processing theory. Social information processing theory claims that the social environment in which individuals are embedded provides a variety of information that affects their attitudes and behaviors, and the individual’s interpretation of this information determines subsequent attitudes and behaviors (Salancik and Pfeffer, 1978). When the organization provides employees via team reflexivity with work-related social information the team is reducing team action errors through a collective reflection of tasks, goals, rules and values, planning, and action/adaptation within the team (Konradt et al., 2016), and achieving control over the overall action, employees would interpret this information and establish a positive perception of organization’s ability and integrity, based on which employee organizational trust is generated accordingly (Salancik and Pfeffer, 1978; Mayer et al., 1995; Rawlins, 2008). Once employees have established trust in the organization, they believe that the organization will honor its commitments, treat them fairly, and have fewer concerns about the risk of being harmed, which would promote the intention to cooperate (Balliet and Van Lange, 2013). According to the above inference, the impact of team reflexivity on employee cooperative behavior is likely to be realized through the establishment of employee organizational trust. Therefore, this study selects organizational trust as the mediating variable of team reflexivity affecting employee cooperative behavior, which is believed to play an important mediating role in the relationship between team reflexivity and employee cooperative behavior.

Furthermore, this study also attempts to further explore the boundary conditions where team reflexivity affects employee cooperative behavior through organizational trust. First, in the process of exerting its effectiveness, team reflexivity may be affected by other organizational environmental factors coexisting with team reflexivity in the organization, such as team context, which may affect the degree of change to individual behavior and attitude brought by team reflexivity (Schippers et al., 2015), while team climate is an important form of team context due to its impacts on employees attitudes and behaviors (Ostroff and Bowen, 2000; Bowen and Ostroff, 2004; Anvuur and Kumaraswamy, 2016). Second, the influence of team reflexivity on organizational trust primarily depends on employees’ processing of social information, however, according to social information processing theory, individuals are more attentive to social information that is relevant to themselves (Salancik and Pfeffer, 1978). Therefore, this relationship is likely moderated by a variable that affects information relevance. By fostering employees’ participation and decision-making power within the work environment (Riordan et al., 2005), employee involvement climate is very likely crucial for connecting employees with their teams thus enhancing the relevance of team-based social information to individuals, which may ultimately influence their capacity to capture information during team reflexivity processes. In summary, we propose employee involvement climate may serve as a significant moderating role in the relationship between team reflexivity and trust. Different from the climates such as teamwork climate (Anvuur and Kumaraswamy, 2016), employee involvement climate emphasizes the attribute of promoting employee self-development by providing employees with decision-making power, encouraging information sharing within the entire organization, training, and rewarding employees based on their job performances (Riordan et al., 2005). It should be noted that teams with a high employee involvement climate help employees increase their self-efficacy, autonomy, and proactivity at work and team processes, thus improving their attention to valuable social information (i.e., strengthening the preference for positive information related to self-development derived from team reflexivity), expand the ability and integrity based social information they can capture from team reflexivity, and improve their organizational trust. In this process, it may influence employees’ perception of the effectiveness and value of team reflexivity. Hence, this study suggests that team reflexivity may positively moderate the relationship between team reflexivity and employee cooperative behavior through organizational trust.

Given all of the above, this research set up a cross-level moderated mediation model to reveal the mechanism of team reflexivity’s influence on employee cooperation behavior and the mediation role of organizational trust in between and look into employee involvement as the boundary condition of the path where team reflexivity indirectly affect employees’ cooperative behavior through organizational trust. The findings may expand our understanding of team reflexivity by explaining organizational trust’s role in team processes, with implications for future research on factors that influence employee cooperative behavior, and provide new ideas and practical guidance for organizations to encourage employees’ cooperative behavior.

## Theory and hypothesis

### Team reflexivity and employee cooperative behavior

Team reflexivity is the degree to which team members openly reflect on team goals, strategies (such as decision-making) and procedures (such as communication) to adapt them to current or expected environmental changes (West, 1996). Team reflexivity is a unique mode of team process and team interaction to promote employee cooperation through the generation of shared mental models through its interactive elements of reflection, planning and action (West, 1996; Marks et al., 2001). First, reflexivity enables team members to exchange their own experience, information and knowledge about the task and goal which helps to form the collective cognitive emergent states (Marks et al., 2001), contributing to the coordination and cooperation (Mohammed et al., 2010). Second, at the action/adaptation phase of reflexivity, the adjustment necessitates the backup behaviors among team members, which signals instrumental and emotional support (Smith-Jentsch et al., 2008), conducive to the generation of cooperative behaviors (Mohammed et al., 2010). Both collective mental states and perceived support the team reflexivity provides would increase the employees’ identification and commitment to the team and improve their cooperation behaviors (Eddy et al., 2013).

In the process of team reflection, team members exchange the knowledge they possess with each other and adjust different perspectives (Swift and West, 1998), in which the sincerity of others’ intention to achieve the team’s goals can be perceived, which can lead individuals to develop an attitude of goodwill preconceptions and trust toward others, thus promoting cooperative behavior toward other employees. On the other hand, through social interaction, individuals can have a deeper understanding of others’ personalities, abilities and other factors, thus forming expectations of others to take cooperative behavior, based on which individuals decide to cooperate or not, since they seek to establish reciprocal relationships (Balliet and Van Lange, 2013; Romano and Balliet, 2017), rather than engage in one-sided exchanges. The process of team reflection, in which members develop shared perceptions about the actual task, generates shared task knowledge related to strategies and goals (Van den Bossche et al., 2011) and can promote commitment to the organization (Schippers et al., 2003), which in turn can increase subjective expectations that others will act cooperatively. In addition, the interactive process of team reflection increases employees’ commitment to team interaction and to some extent indicates their attitudes and willingness to cooperate. Thus, employees will choose to cooperate because of the expectation that their colleagues will adopt cooperative behavior. Based on the above information, we propose the following hypothesis:

H1: Team reflexivity is positively related to employee cooperative behavior.

### The mediating role of organizational trust

Organizational trust is employees’ willingness to be vulnerable to the organization based on the confidence in the organization’s ability, integrity, and benevolence through social interactions, as well as on the perception of safety and reliability derived from that confidence (Nyhan and Marlowe Jr, 1997; Dirks and Ferrin, 2002). During the process of team reflexivity, employees engage in extensive social interactions, generating abundant social information, which is a core element of social information processing theory. According to this theory, the interpretation of such social information can influence employees’ subsequent attitudes and behaviors (Salancik and Pfeffer, 1978). Moreover, as we have noted, trust is cultivated through social interactions. Thus, we argue that social information processing theory likely elucidates the theoretical mechanism by which team reflexivity fosters organizational trust. Namely, employees can capture organization-related social information during their participation in social interactions within team reflexivity, and their processing and interpretation of this information subsequently enhance their organizational trust. Specifically, the cyclic phases of reflection, planning, and action/adaptation of team reflexivity can continuously reduce the team’s action errors, keep the overall actions under control (Konradt et al., 2016), to achieve team goals and improve performance (Vashdi et al., 2007; Tannenbaum and Cerasoli, 2013). Therefore, individuals can have a positive appraisal of the working ability based on reflection, error correction, and execution of colleagues participating in each phase of team reflexivity, and further process and assimilate such appraisal and the information provided by colleagues into the judgment about their overall working environment, to generate organizational trust based on the ability of organization and leaders (Mayer et al., 1995; Dirks and Ferrin, 2002), for example, they hold a belief that the organization and leaders have the ability to lead them on the right development path, achieve greater goals and create better performance. Specifically, at the levels of shallow and moderate reflection which are more focused on the task and work process (Swift and West, 1998), employees tend to generate ability-based organizational trust. Additionally, at the deep level of reflection, team members would openly discuss the team’s norms and values (Swift and West, 1998). This process can help employees obtain relevant information about not only the content of team norms and values but also the team’s reflection and action adjustment on norms and values, which conveys the message that the team attaches importance to norms and values and operates based on them. This provides the basis for employee’s appraisal of the integrity of the organization and helps employees to form integrity-based trust in the organization. Therefore, we can infer that when employees participate in the process of team reflexivity and receive the team’s work-related social information about tasks, goals, strategies, norms and values, etc., the level of organizational trust will increase.

Organizational trust also has an important impact on employee cooperative behavior. According to information processing theory, individuals make decisions based on their understanding and interpretation of processed information (Simon, 1978), when employees trust in the organization, they will assume that the organization will treat them honestly and fairly and has their best interests at heart (Mayer et al., 1995), which reduces employees’ concern on the possible negative consequences of cooperative behavior. As a result, they are more willing to act cooperatively because they trust the organization to protect their interests. In addition, trust facilitates information sharing (Özer et al., 2011), employees are less worried that sharing critical information about projects, problems, or opportunities would be misused or used against them. Instead, they are more willing to share that information because they trust that the organization and their colleagues will use it wisely to advance cooperation and common goals, and then are more actively engaged in cooperative behavior. Therefore, this study proposes that organizational trust might take effect as a mediator on the relationship between team reflexivity and employee cooperation behavior, so we hypothesize:

H2: Organizational trust mediates the relationship between team reflexivity and employee cooperative behavior.

### The moderating role of employee involvement climate

Employee involvement climate refers to a climate of involvement in terms of employee perceptions of participative decision-making, information sharing, performance-based rewards, and training in a work environment that all employees can recognize (Riordan et al., 2005). Research has demonstrated that the employee involvement climate helps to facilitate employees with enough authority, more autonomy, skills for mutual helping, and common norms to shape the individual positive psychological environment for cooperative behavior and to create a positive cooperative climate (van Gerwen et al., 2018b; Chiang and Chen, 2021). Therefore, this study considers employee involvement climate as a contextual factor affecting the previously hypothesized relationship between team reflexivity, employee cooperative behavior, and organizational trust. This study proposes that the relationship between team reflexivity and employees’ organizational trust is significantly enhanced under a high level of employee involvement. As mentioned in H2 above, team reflexivity can convey information about organizational ability and integrity through the paths of reducing errors, improving performance, and reflecting on values, so as to promote employees’ sense of organizational trust. The autonomy given to employees by a high involvement climate represents the trust of management in employees, based on the principle of reciprocity, a high involvement climate will also enhance employees’ trust in the organization. However, a low employee involvement climate means that employees’ work autonomy, self-efficacy and sense of control are all at a relatively low-level (Parker et al., 1994; Riordan et al., 2005), employees cannot perceive the importance of their work easily, their sense of involvement and enthusiasm are weakened, and their level of exchange with the organization, leaders and members is correspondingly lower. On one hand, employees’ attention to the information provided by team reflexivity will be weakened, based on the social information processing theory, the process of information processing about organizational error correction, execution ability, and persistent principles is weakened, accordingly, employees’ trust in colleagues, leaders and organizations based on ability and integrity will also be weakened; On the other hand, when EI climate is low, employees may have a sense of alienation outside the core of the organization and generate the “outsider” identity (Stamper and Masterson, 2002), even though team reflexivity strives to include every member in the team’s reflection and discussion (Wang et al., 2021), team reflexivity may be more regarded as a routine process by the employees without the genuine engagement. Therefore, a low employee involvement climate hedges and weakens the positive effects of team reflexivity on trust and belonging in the organization. Instead, an employee involvement climate with the feature of information sharing would provide employees with more social information related to the ability and integrity of the team to generate the ability and integrity-based trust. Additionally, an employee involvement climate also enables employees to have a stronger sense of autonomy and involvement in work, the team reflexivity would maximize the acceptance of information collected and processed by the employees to increase their trust in teams and organization. Therefore, the promotion effect of team reflexivity on organizational trust is strengthened by a high employee involvement climate. Given the analysis, we propose the following hypothesis:

H3: Employee involvement climate moderates the relationship between team reflexivity and organizational trust such that this relationship is stronger when employee involvement climate is high than when it is low.

### Moderated mediation hypothesis

Based on a combination of H2 and H3, this study further proposes a moderated mediation model. When employees are in a low involvement climate, they will feel less autonomy and enthusiasm in work, and feel difficult and unwilling to deeply participate in the team reflexivity process to communicate and interact with others (i.e., poor perception and reception of social information about the ability and integrity of the organization), thus they are less likely to generate their organizational trust. Employees who have a low level of organizational trust, lack the belief that the organization will treat them fairly and protect their rights and interests, instead, they are more likely to be concerned that their rights and interests will be harmed and that the critical information they share about their work might be misused or hostilely used, becoming less likely to cooperate. When employees are in a high-involvement climate, they will be more eager to achieve self-development through work and expect effective information feedback. In this way, they will perform a stronger ability of information capturing (i.e., capturing more ability-and-integrity-based information) in the process of team reflexivity. At the same time, they are more likely to feel respected and trusted through the power of work autonomy granted by the organization. With such psychological safety, they are more willing to share information and cooperate. Therefore, employee involvement climate moderates the indirect effects of team reflexivity on employees’ cooperative behavior through organizational trust. We thus propose:

H4: Employee involvement climate moderates the indirect effect of team reflexivity on employee cooperative behavior through the mediating effect of organizational trust such that when the level of employee involvement climate is high, the indirect effect is stronger than when it is low.

**Figure 1:**
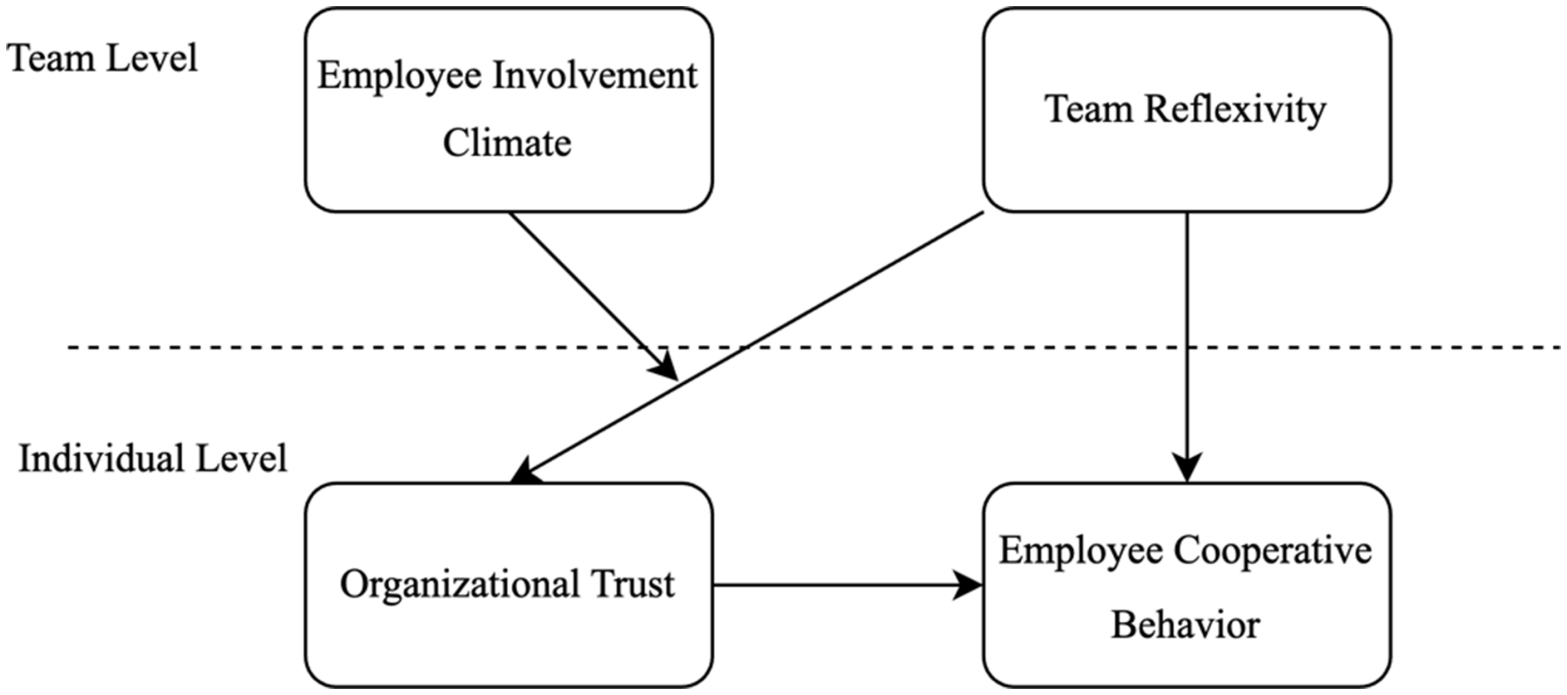
Conceptual model of this research.

Based on the above theories and hypotheses, the conceptual model of this study is shown in Figure 1.

## Research methods

### Sample and data collection

In this study, we collected data from 84 project teams in different companies in Shandong province and Beijing in China. A questionnaire was administered to 504 employees from these 84 teams through the snowball sampling method, which the employees completed on an online platform covering team reflexivity, employee involvement, organizational trust, employee cooperative behavior, and demographic characteristics were administered to 504 employees from the 84 teams by the snowball sampling method. All respondents completed the questionnaire voluntarily and were assured that their responses would be kept confidential. After eliminating questionnaires with overly consistent answers and questionnaires with apparently contradictory reverse question tests, a total of 412 valid questionnaires were returned, 412 valid questionnaires were returned with a return rate of 81.7%. In the sample, the average team size is five members, 49.4% were male, 49.9% were aged 24–28, 50.1% were aged 29–33, and 72.1% had a bachelor’s degree or above. In terms of occupation, the participants mainly work in institutions/state enterprises and other types of enterprises (such as private enterprises and foreign enterprises), accounting for 27.1 and 52.1% respectively, while 16.7% are self-employed and 4.1% are others. In terms of tenure, 16.2% of the respondents have worked for 2 years or less, 64.87% have worked for 3–9 years, and 18.9% have worked for more than 10 years. Most of the respondents were junior employees of the company, accounting for 58.4%, followed by junior managers accounting for 36.3%, and middle and senior managers, accounting for a relatively small proportion of 5.3%. In terms of the type of department, the participants were mainly in the business and technical categories, accounting for 37.1 and 32.7% respectively, while the management category accounted for 18.4% and the others accounted for 11.9%.

Since the data in this study are all from employees’ self-rating, there may be common method variance. Therefore, this study uses Harman’s one-factor test for the common method variance test, and the variance explained by the first factor before rotation is 29.01%, so it can be concluded that there is no common method variance in the sample data.

### Measurements

#### Team reflexivity

Carter and West (1998) originally developed a 16-item team reflexivity scale, encompassing two dimensions: task reflexivity and social reflexivity. However, the adequacy of social reflexivity in reflecting team reflexivity remains debated. Many scholars typically focus solely on the task reflexivity dimension in empirical studies, suggesting that social reflexivity is not yet a stable and measurable construct. Accordingly, we adopted the 5-item scale adapted by De Jong and Elfring (2010) from the Carter and West scale (Carter and West, 1998), following the precedent set in existing literature (Yang et al., 2020; Ye et al., 2021). This adapted scale has demonstrated excellent reliability and validity in prior studies (Leblanc et al., 2022; Santos and Neumeyer, 2022), and effectively captures the construct of team reflexivity. Items were measured on a five-point scale ranging from 1 = “Strongly disagree” to 5 = “Strongly agree,” Sample items include: “The team often reviews its objectives,” “We regularly discuss whether the team is working effectively together,” and the Cronbach’s alpha is 0.89. As team reflexivity is a team-level construct, according to Chan’s typology of composition models, we adopted the additive composition approach, which involved averaging across individual team members’ team reflexivity perceptions, regardless of within-team variability in those perceptions (Chan, 1998). To test the validation of data aggregation, we calculated the ICC (intra-group correlation coefficient) and RWG (within-group interrater reliability) values: ICC (1) = 0.62, ICC (2) = 0.89, RWG = 0.87, which support the aggregation of individual scores used for team-level analysis (James et al., 1984; LeBreton and Senter, 2008).

#### Organizational trust

Organizational trust was assessed with four items based on Rawlins (2008). The scale consists of four parts: overall trust, the organization shows competence, the organization shows integrity, and the organization shows goodwill. All four items were measured on a 5-point scale ranging from 1 = “Strongly disagree” to 5 = “Strongly agree.” The questions included “I trust the organization to take care of people like me” and “This organization has the ability to accomplish what it says it will do. “Cronbach’s alpha value of the scale was 0.86, which confirmed the internal reliability of the measures used (Nunnally and Bernstein, 1994).

#### Employee involvement climate

For the measurement of employee involvement climate, the 18-item scale of Riordan et al. was selected, which includes four dimensions: power sharing, information sharing, rewards and recognition system, knowledge development, and training (Riordan et al., 2005). The questions included “I have sufficient authority to fulfill my job responsibilities,” “I receive sufficient training to do my job,” and so on. All were measured on a five-point scale ranging from 1 = “Strongly disagree” to 5 = “Strongly agree.” The Cronbach’s alpha value for the scale was 0.93, and the validation of data aggregation results were: ICC (1) = 0.68, ICC (2) = 0.91, and RWG = 0.91. all measured on a five-point scale ranging from 1 = “Strongly disagree” to 5 = “Strongly agree.” These results showed the appropriateness of data aggregation.

#### Employee cooperative behavior

Simmons et al. developed the cooperation and competition strategy scale (Simmons et al., 1988), which consists of two subscales: competition and cooperation. Eleven items were used to measure competitive behavior and eight items were used to measure cooperative behavior. This study adopted the items that measured cooperative behavior, including “Individual success can be achieved while working with others,” “I enjoy working with others to achieve joint success” and so on, all measured on a five-point scale ranging from 1 = “Strongly disagree” to 5 = “Strongly agree.” The Cronbach’s alpha value for the scale was 0.92.

#### Control variables

We also controlled for individuals’ age, gender, education level, and tenure. Because previous research suggested that these individual variables might be correlated (Ayoko, 2016; Fu et al., 2019). In addition, because team size is an important structural variable and has potential impacts on team members’ cooperative willingness (Hamburger et al., 1975; Hoegl, 2005), our study considered team size as a control variable.

## Results

### Confirmatory factor analysis

We conducted a series of confirmatory factor analysis (CFA) to compare our hypothesized four-factor model (team reflexivity, organizational trust, employee involvement, and employee cooperative behavior) to a series of alternative models, as it allows us to verify that the hypothesized relationships provide the best fit for the data. Table 1 presents the CFA results. The results indicated that the four-factor model fitted the data well (*χ*^2^ = 116.50, df = 372, *χ*^2^/df = 0.31, CFI = 0.98, TLI = 0.98, RMSEA = 0.06, SRMR = 0.04). The *χ*^2^/df ratio of 0.31, which falls well below the commonly accepted threshold of 3.0 (Kline, 2015). The model performed better than alternative models—including a three-factor model in which organizational trust and employee cooperative behavior were loaded onto one factor; a two-factor model in which team reflexivity, organizational trust, and employee cooperative behavior were loaded onto one factor; and a one-factor model in which all items were loaded onto one factor.

**Table 1:** Results of confirmatory factor analysis.

Measurement model	*χ* ^2^	df	CFI	TLI	RMSEA	SRMR
Hypothesized four-factor model	116.50	372	0.98	0.98	0.06	0.04
Three-factor model	261.70	375	0.97	0.96	0.07	0.03
Two-factor model	1,136.30	377	0.89	0.87	0.09	0.05
One-factor model	15,254.60	378	0.40	0.22	0.31	0.05

*χ2, Chi-square; df, degree of freedom; CFI, comparative fit index; TLI, Tucker–Lewis index; RMSEA, root-mean-square error of approximation; SRMR, standardized root mean square residual.*

### Descriptive statistics and correlations

Table 2 presents the results of the correlation analysis. As shown in the table team reflexivity was positively related to both organizational trust (*r* = 0.35, *p* < 0.001) and employee cooperative behavior (*r* = 0.24, *p* < 0.001), and organizational trust was positively related to employee cooperative behavior (*r* = 0.49, *p* < 0.001). These results provide initial support for some of our hypotheses.

**Table 2:** Descriptive statistics and intercorrelations.

	Mean	SD	1	2	3	4	5	6	7	8
1. Gender	0.50	0.50								
2. Age	0.50	0.50	−0.08							
3. Education level	4.03	0.97	0.03	0.02						
4. Tenure	2.56	0.98	−0.08	0.55**	0.03					
5. TR	3.65	0.81	0.06	0.01	0.06	0.00				
6. EI	3.60	0.76	−0.06	0.01	0.05	0.02	0.21**			
7. OT	3.86	0.78	−0.01	0.08	0.17**	0.04	0.35**	0.46**		
8. ECB	3.70	0.88	−0.03	−0.05	0.13**	0.01	0.24**	0.39**	0.49**	
9. Team size	5.08	0.87	0.00	−0.05	0.01	−0.01	−0.01	0.16**	0.14**	0.18**

***p < 0.001, **p < 0.01, *p < 0.05.

SD, standard deviation; TR, team reflexivity; EI, employee involvement; OT, organizational trust; ECB, employee cooperative behavior.

### Hypotheses testing

**Figure 2:**
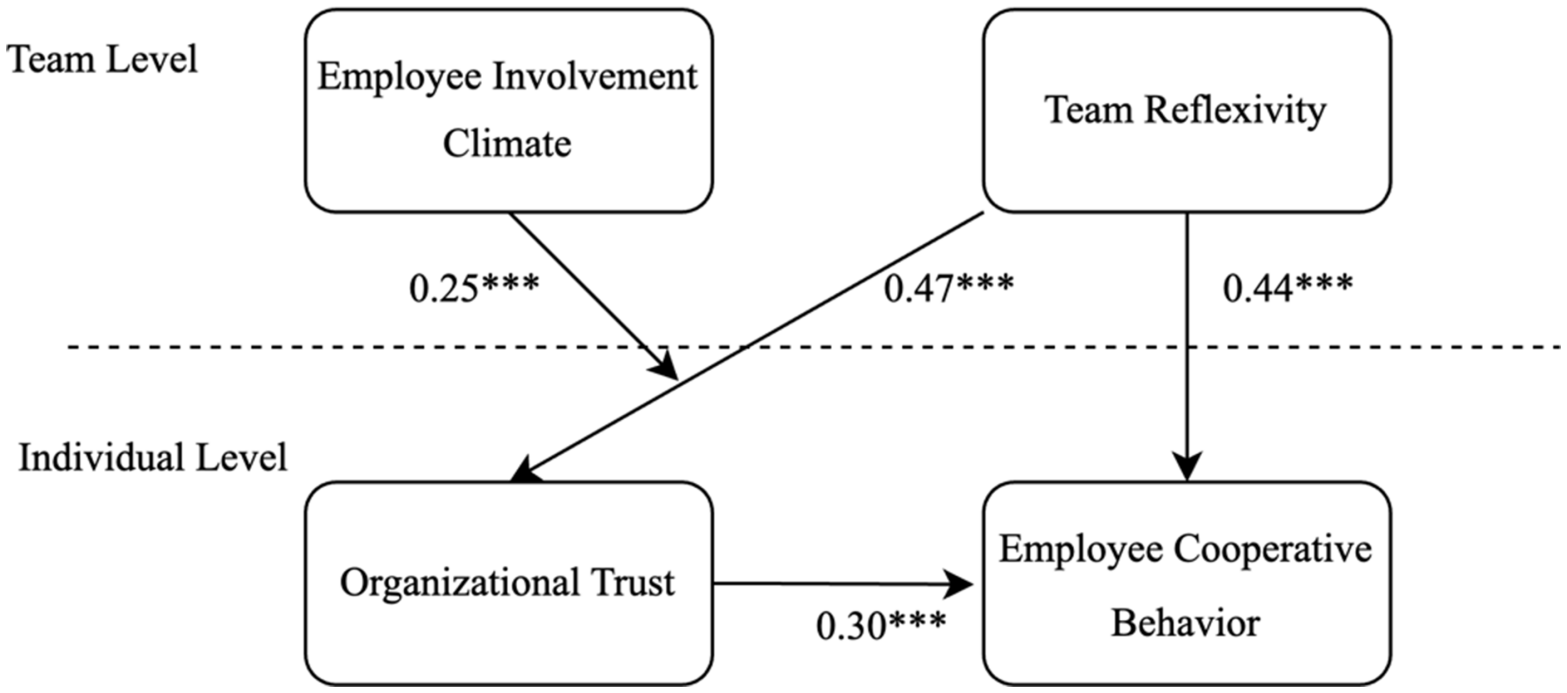
Multilevel SEM model path analysis. ***p < 0.001, **p < 0.01, *p < 0.05.

Figure 2 shows the path coefficients between the variables in this study. We tested all the hypothesized relationships simultaneously using Stata 16. Table 3 summarizes the results. H1 predicted that team reflexivity is positively related to employee cooperative behavior. Findings provide empirical evidence that shows the significant effect of team reflexivity on employee cooperative behavior (*r* = 0.41, *p* < 0.001). Thus, H1 is supported. H2 predicted that organizational trust mediates the relationship between team reflexivity and employee cooperative behavior. To examine this mediation effect, we followed the multilevel mediational model proposed by Krull and MacKinnon (2001). The result shows that team reflexivity was significantly associated with organizational trust (*r* = 0.47, *p* < 0.001), and the mediator was significantly related to employee cooperative behavior (B = 0.30, *p* < 0.001) when the predictor variable team reflexivity was in the model (*r* = 0.44, *p* < 0.001, Model 3). This result suggests that organizational trust partially mediated the relationship between team reflexivity and employee cooperative behavior. Bootstrapping of the sampling distribution was also conducted regarding the indirect effect. The results showed that the 95% confidence interval of the indirect effect was between 0.17 and 0.49. Thus, H2 was supported.

**Table 3:** Results of hypotheses testing.

Variables	Model 1	Model 2	Model 3	Model 4
	ECB	OT	ECB	OT
Individual level (*N* = 412)				
OT			0.30***	0.26***
			(0.05)	(0.06)
Team level (*N* = 84)				
TR	0.41***	0.47***	0.44***	0.51***
	(0.01)	(0.08)	(0.10)	(0.07)
EI				0.19***
				(0.05)
TR*EI				0.25***
				(0.08)
Observations	412		412	412
Number of groups	84		84	84

Standard errors in parentheses, ***p < 0.001, **p < 0.01, *p < 0.05.

TR, team reflexivity; EI, employee involvement; OT, organizational trust; ECB, employee cooperative behavior.

H3 predicted that employee involvement moderates the relationship between team reflexivity and organizational trust. As shown in Table 3, the interaction of team reflexivity and Employee Involvement was significant when the dependent variable is organizational trust (*γ* = 0.25, *p* < 0.001). Employee Involvement is a moderator between team reflexivity and organizational trust. Thus, H3 is supported.

**Figure 3:**
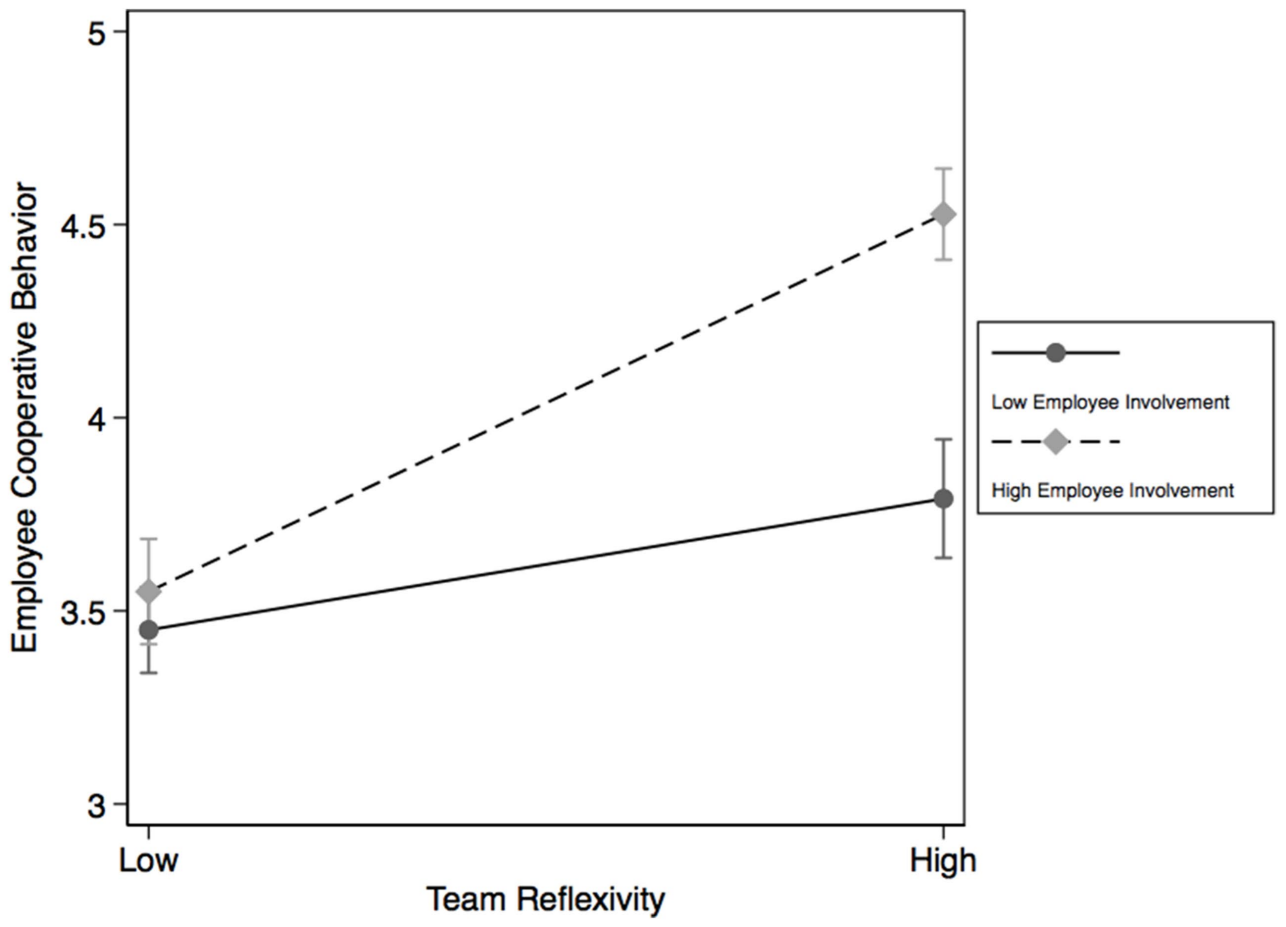
Simple slope for the interaction effect of team reflexivity and employee involvement on organizational trust.

As shown in Figure 3, the simple slope test results show that when employees have a higher level of Involvement, team reflexivity has a stronger effect on employee cooperative behavior. When employee involvement is low, team reflexivity has a relatively weak effect on employee cooperative behavior. Based on this, hypothesis 3 is verified again.

Furthermore, this study used the method proposed by Preacher et al. to test the moderated mediation effect (Preacher et al., 2007). As shown in Table 4, the bootstrapping results showed that the mediating effect of organizational trust on employee cooperative behavior was 0.46, 95% CI = [0.24, 0.67]. When the level of employee involvement is high, the indirect effect is 0.52, 95% CI = [0.27, 0.77] When the level of employee involvement level is low, the indirect effect is 0.39, 95% CI = [0.21, 0.57]. Therefore, Hypothesis 4 was supported.

**Table 4:** Moderated mediation effect analysis result.

Level	Indirect effect	SE	95% Confidence interval	
High	0.52***	0.13	0.27	0.77
Low	0.39***	0.09	0.21	0.57
Indirect effect difference	0.46***	0.11	0.24	0.67

***p < 0.001, **p < 0.01, *p < 0.05.

SE, standard errors.

## Discussion

### Result discussion

The cooperative behavior of employees is one of the key elements for organizations to reduce risks, cope with challenges and increase competitive advantages in an uncertain and competitive environment. Although a lot of literature has helped us understand the sources of cooperative behavior from various levels, there is still a lack of understanding of the mechanism affecting employee cooperative behavior from the perspective of team process and organizational climate. This study aims to explore the relationship between team reflexivity and employee cooperative behavior, along with the mediating effect of employees’ organizational trust, and the moderating effect of the involvement climate perceived by employees. Based on social information processing theory, this study establishes a cross-level moderated mediation model and obtains three key results through data analysis.

First, our results show that team reflexivity, as a contextual factor at the team level, has a significantly positive impact on employee cooperative behavior at the individual level. The reflection phase of team reflexivity helps to promote the interaction process such as the exchange of experience and knowledge among employees so that employees are able to obtain an accurate and shared understanding of the current work, goals, and even team values, and further promote employees’ cooperative behavior at the individual level, which is consistent with the previous literature (Mohammed et al., 2010; Konradt et al., 2015; Konradt et al., 2016). The planning phase of team reflexivity helps team members co-design their work, thereby reducing overall team demands, enhancing effectiveness, and promoting team collaboration at the task level, which is also in line with the scholars’ research (Schippers et al., 2003; Ellis et al., 2010). The action/adaptation process provides members with backup behavior, as well as the management of member conflicts that may occur at any point in time, especially for the new generation of employees who are emotional (Hou et al., 2014), which helps the team convey their instrumental and emotional support to members, and establish a supportive and cohesive climate that stimulates cooperative behavior (Marks et al., 2001; Smith-Jentsch et al., 2008).

Secondly, the results also show that the level of employees’ organizational trust mediates the relationship between team reflexivity and employee cooperative behavior. This shows that team reflexivity can further promote employee cooperative behavior by promoting organizational trust, team reflexivity has a positive impact on organizational trust, which in turn has a positive effect on employee cooperative behavior. Team reflexivity reduces the gap between the actual task completion and the task goal through the process of task reflexivity, and navigates the team and members on the correct track as much as possible, such correctness helps to meet the needs of the new generation of employees to pursue their personal value (Hou et al., 2018), stimulates their perception of the safety and reliability of the environment, and promotes the generation of organizational trust. This echoes the definition of organizational trust by Dirks and Ferrin (2002). Simultaneously, team reflexivity reflects the organization’s ability, such as the ability to reflect on the complete team process, correct errors, execute the plans, and improve the team performance; When reflecting on the team rules and values, team reflexivity conveys the message that the organization is making effort to ensure that its actions are consistently based on its norms and values, which shows the integrity of the organization. Thus, team reflexivity promotes the information captured by employees about the ability and integrity of the organization, contributing to the generation of organizational trust. Our results are in line with existing research (Ferrin et al., 2008; Balliet and Van Lange, 2013; Peñarroja et al., 2013), once employees raise their trust in the organization, they perform a high level of cooperation. On this basis, organizational trust plays a mediating role, closely linking the relationship between team process and individual behavior.

Finally, employee involvement climate has a significant moderating effect on the relationship between team reflexivity and organizational trust and further promotes the indirect relationship between team reflexivity and cooperation. This means that when employees are in an organization with a high involvement climate, they can better feel the importance of their own work, their sense of involvement and enthusiasm will be correspondingly higher, and the will of self-development and self-realization will be stimulated (Mohrman and Lawler, 1989), which is exactly what the new generation of employees are striving for (Chen and Zhou, 2018; Hou et al., 2018). Therefore, the new generation employees will have more expectations to obtain information conducive to self-development through the process of team reflexivity, so that they are more proactive to immerse themselves in the process of team reflexivity, perceive a stronger sense of value and emotional support, generate a stronger sense of organizational trust (Dirks and Ferrin, 2002; Chen and Zhou, 2018), and improve their willingness to participate in information sharing and cooperation in such a psychologically safe environment. This finding confirms the expectations of the new generation employees for feedback and self-development, thus it responds to the related literature (Kim et al., 2018), and further develops the literature by providing a mechanism that takes both feedback (i.e., team reflexivity) and self-development (i.e., employee involvement climate) as well as their interaction into consideration to understand how to deal with the reluctance of cooperation of the new generation employees.

### Theoretical implications

The theoretical contribution of this study is twofold. First, we develop a cross-level moderated mediation model to reveal the mechanism of team reflexivity’s influence on employee cooperative behavior. The results show that team reflexivity performance can promote employees’ organizational trust, and then positively promote cooperative behavior, confirming the previous literature opinions (Mohammed et al., 2010; Konradt et al., 2015). More importantly, it provides evidence supporting the positive social and interpersonal effect of task reflexivity, which has not been adequately recognized before. Specifically, task reflexivity (which is used as the measurement of team reflexivity in most reflexivity research) is commonly regarded as impacting team effectiveness and performance (Konradt et al., 2015; Fu et al., 2019), while the interpersonal effect of the task reflexivity is usually left unexamined. The result of this study demonstrates that task reflexivity could also affect employee’s trust in the team, which in turn affects employees’ cooperation attitude and behavior.

Secondly, in response to the study of employee cooperation within a team from the perspective of interaction, we conducted this research from the perspective of reflexivity, which enriched our understanding of the generating process of employee cooperative behavior. Given employee cooperative behavior is an adaptive and dynamic process, scholars call for the need to continue to expand the understanding of the key factors of it (Salas et al., 2015), in order to further understand the process of cooperation (Driskell et al., 2018), that is, how team interaction promotes cooperation from the perspective of the interaction mechanism within the team. Currently, there is some research on cooperation from the perspective of team interaction, but little research on cooperation from the perspective of team reflexivity; additionally, the study identifies the interaction effect of employee involvement climate and team reflexivity, indicating that organizational climate and team process could jointly affect employee trust and cooperation behavior, contributing to more understanding of the cooperation behaviors in the context of the organizations.

### Practical implications

The study has several practical implications. First of all, in terms of team management, managers should carry out a long-term, regular, and cyclic team reflexivity process from shallow to deep, focusing on disclosing and emphasizing more information related to team ability and integrity in this process to increase employees’ trust. Team reflexivity can enhance team members’ sense of belonging and cooperative behavior, reduce adverse competition within the team, and encourage team members to help each other jointly cope with work challenges and performance pressure. In addition, team reflexivity can not only promote employees to be more altruistic and team spirit oriented, but more importantly, motivate those egoistic and self-centered new generation of post-90s employees to actively improve their work performance through clearly capturing and interpreting positive social information in the process of team reflexivity regardless of personality type, thus enhancing their organizational trust and becoming more committed to the work and cooperative toward goals. Finally, for organizational development, it is important to promote an interactive process of team reflexivity and a climate of employee involvement. To effectively achieve performance, only the superficially stylized team interaction is not enough, especially given the situation that the current generation of post-90s employees tend to be self-centered, in this case, it is difficult for them to actively participate in the interaction process with others and the team to achieve the team goal. Therefore, organizations need to urge a working climate, for example, implementing regular team reflection sessions where employees can openly discuss past projects or creating open communication platforms (e.g., an anonymous feedback system or direct access to leaders) where employees can share their thoughts on company processes, decisions or strategies, increases employee motivation and initiative to participate in the team reflection process, and improves employees’ trust in the organization, which in turn fosters collaboration and organizational performance more effectively.

## Limitations and future research

The study also has some limitations, which we hope to further explore in future research. First, this study uses cross-sectional data and cannot prove a causal relationship between team reflexivity and employee cooperative behavior. In the future, multiple waves of data could be collected for longitudinal studies to verify causality. Second, due to the limitation of time and resources, this study only used data from two provinces in China, which cannot verify the universality of the conclusions of this study to other cultural backgrounds. We encourage future studies to replicate and extend this research in diverse settings to enhance the external validity of the results. Third, while we focused on organizational trust as a mediator, our empirical results indicate that it only partially mediates the relationship between team reflexivity and employee cooperative behavior. This suggests the presence of other mediating mechanisms. Future research could explore additional mediators, such as job satisfaction, psychological safety, or workload (Schippers et al., 2015), to provide a more comprehensive understanding of the underlying processes.

## Data Availability

The raw data supporting the conclusions of this article will be made available by the authors without undue reservation.

## References

[ref1] Adler N. J. Gundersen A. (2001). International dimensions of organizational behavior. Cincinnati, OH: South-Western.

[ref2] Amirtharaj B. Cross K. Vembar V. (2011). Role of training and development in promoting the growth of hospitality industry. Int. J. Manag. (IJM) 2, 126–133.

[ref3] Anvuur A. M. Kumaraswamy M. M. (2016). Effects of teamwork climate on cooperation in crossfunctional temporary multi-organization workgroups. J. Constr. Eng. Manag. 142:04015054. doi: 10.1061/(ASCE)CO.1943-7862.0001029

[ref4] Arumsari D.N. Rini H.P. Cahyani N.M. Kosay P. (2023). "Cooperatives employee performance base on competence and training", in: Proceedings of international conference on economics business and government challenges, 198–205.

[ref5] Ayoko O. B. (2016). Workplace conflict and willingness to cooperate: the importance of apology and forgiveness. Int. J. Confl. Manag. 27, 172–198. doi: 10.1108/IJCMA-12-2014-0092

[ref6] Balliet D. Van Lange P. A. (2013). Trust, conflict, and cooperation: a meta-analysis. Psychol. Bull. 139, 1090–1112. doi: 10.1037/a0030939 23231532

[ref7] Barrick M. R. Mount M. K. (1991). The big five personality dimensions and job performance: a meta-analysis. Pers. Psychol. 44, 1–26. doi: 10.1111/j.1744-6570.1991.tb00688.x

[ref8] Blau P. (2017). Exchange and power in social life. New York: Routledge.

[ref9] Bowen D. E. Ostroff C. (2004). Understanding HRM–firm performance linkages: the role of the “strength” of the HRM system. Acad. Manag. Rev. 29, 203–221. doi: 10.2307/20159029

[ref10] Byrne J. A. (1993). The horizontal corporation. Bus. Week 20, 76–81.

[ref11] Cameron L. Erkal N. Gangadharan L. Meng X. (2013). Little emperors: behavioral impacts of China's one-child policy. Science 339, 953–957. doi: 10.1126/science.1230221, PMID: 23306438

[ref12] Carter S. M. West M. A. (1998). Reflexivity, effectiveness, and mental health in BBC-TV production teams. Small Group Res. 29, 583–601. doi: 10.1177/1046496498295003

[ref13] Chan D. (1998). Functional relations among constructs in the same content domain at different levels of analysis: a typology of composition models. J. Appl. Psychol. 83, 234–246. doi: 10.1037/0021-9010.83.2.234

[ref14] Chang P.-C. Ma G. Lin Y.-Y. (2022). Inclusive leadership and employee proactive behavior: a cross-level moderated mediation model. Psychol. Res. Behav. Manag. 15, 1797–1808. doi: 10.2147/PRBM.S363434, PMID: 35860206 PMC9292059

[ref15] Chatman J. A. Barsade S. G. (1995). Personality, organizational culture, and cooperation: evidence from a business simulation. Adm. Sci. Q. 40, 423–443. doi: 10.2307/2393792

[ref16] Chen Y. He X. Lu L. Gao X. (2022). In a team forgiveness climate, the influence of paradoxical thinking of leaders on the team voice behavior: mediated by team cooperation. PLoS One 17:e0265018. doi: 10.1371/journal.pone.0265018, PMID: 35290375 PMC8923504

[ref17] Chen M. Zhou S. (2018). Research on the impact of participatory management on the loyalty of new generation employees: a modeled mediating effect model. Indus. Technol. Econ 37, 12–18. doi: 10.3969/j.issn.1004-910X.2018.10.002

[ref18] Chiang C.-F. Chen J.-A. (2021). How empowering leadership and a cooperative climate influence employees’ voice behavior and knowledge sharing in the hotel industry. J. Qual. Assur. Hosp. Tour. 22, 476–495. doi: 10.1080/1528008X.2020.1802391

[ref19] De Jong B. A. Elfring T. (2010). How does trust affect the performance of ongoing teams? The mediating role of reflexivity, monitoring, and effort. Acad. Manag. J. 53, 535–549. doi: 10.5465/amj.2010.51468649

[ref20] Dirks K. T. Ferrin D. L. (2002). Trust in leadership: meta-analytic findings and implications for research and practice. J. Appl. Psychol. 87, 611–628. doi: 10.1037/0021-9010.87.4.611, PMID: 12184567

[ref21] Driskell J. E. Salas E. Driskell T. (2018). Foundations of teamwork and collaboration. Am. Psychol. 73, 334–348. doi: 10.1037/amp0000241, PMID: 29792452

[ref22] Eddy E. R. Tannenbaum S. I. Mathieu J. E. (2013). Helping teams to help themselves: comparing two team-led debriefing methods. Pers. Psychol. 66, 975–1008. doi: 10.1111/peps.12041

[ref23] Edmondson A. C. (2002). The local and variegated nature of learning in organizations: a group-level perspective. Organ. Sci. 13, 128–146. doi: 10.1287/orsc.13.2.128.530, PMID: 19642375

[ref24] Ellis S. Ganzach Y. Castle E. Sekely G. (2010). The effect of filmed versus personal after-event reviews on task performance: the mediating and moderating role of self-efficacy. J. Appl. Psychol. 95, 122–131. doi: 10.1037/a0017867, PMID: 20085410

[ref25] Fang Y.-C. Chen J.-Y. Wang M.-J. Chen C.-Y. (2019). The impact of inclusive leadership on employees’ innovative behaviors: the mediation of psychological capital. Front. Psychol. 10:1803. doi: 10.3389/fpsyg.2019.01803, PMID: 31447740 PMC6691172

[ref26] Ferrin D. L. Bligh M. C. Kohles J. C. (2008). It takes two to tango: an interdependence analysis of the spiraling of perceived trustworthiness and cooperation in interpersonal and intergroup relationships. Organ. Behav. Hum. Decis. Process. 107, 161–178. doi: 10.1016/j.obhdp.2008.02.012

[ref27] Fu K.-J. Hsieh J.-Y. Wang T. K. (2019). Fostering employee cooperation behavior in the federal workplace: exploring the effects of performance management strategies. Public Pers. Manag. 48, 147–178. doi: 10.1177/0091026018801038

[ref28] Gilbert J. A. Carr-Ruffino N. Ivancevich J. M. Konopaske R. (2012). Toxic versus cooperative behaviors at work: the role of organizational culture and leadership in creating community-centered organizations. Int. J. Leadersh. Stud. 7, 29–47.

[ref29] Goh E. Lee C. (2018). A workforce to be reckoned with: the emerging pivotal generation Z hospitality workforce. Int. J. Hosp. Manag. 73, 20–28. doi: 10.1016/j.ijhm.2018.01.016

[ref30] Gursoy D. Maier T. A. Chi C. G. (2008). Generational differences: an examination of work values and generational gaps in the hospitality workforce. Int. J. Hosp. Manag. 27, 448–458. doi: 10.1016/j.ijhm.2007.11.002

[ref31] Hamburger H. Guyer M. Fox J. (1975). Group size and cooperation. J. Confl. Resolut. 19, 503–531. doi: 10.1177/002200277501900307

[ref32] Hoegl M. (2005). Smaller teams–better teamwork: how to keep project teams small. Bus. Horiz. 48, 209–214. doi: 10.1016/j.bushor.2004.10.013

[ref33] Hou X. Li Y. Tu Y. (2014). Work values of Chinese millennial generation: structure, measurement and effects on employee performance. Acta Psychol. Sin. 46:823. doi: 10.3724/SP.J.1041.2014.00823

[ref34] Hou X. Li W. Yuan Q. (2018). Frontline disruptive leadership and new generation employees’ innovative behaviour in China: the moderating role of emotional intelligence. Asia Pac. Bus. Rev. 24, 459–471. doi: 10.1080/13602381.2018.1451126

[ref35] Huang Y. Fan D. Su Y. Wu F. (2018). High-performance work systems, dual stressors and ‘new generation’ employee in China. Asia Pac. Bus. Rev. 24, 490–509. doi: 10.1080/13602381.2018.1451127

[ref36] James L. R. Demaree R. G. Wolf G. (1984). Estimating within-group interrater reliability with and without response bias. J. Appl. Psychol. 69, 85–98. doi: 10.1037/0021-9010.69.1.85

[ref37] Kim M. Choi L. Borchgrevink C. P. Knutson B. Cha J. (2018). Effects of Gen Y hotel employee’s voice and team-member exchange on satisfaction and affective commitment between the US and China. Int. J. Contemp. Hosp. Manag. 30, 2230–2248. doi: 10.1108/IJCHM-12-2016-0653

[ref38] Kline P. (2015). A handbook of test construction (psychology revivals): introduction to psychometric design. New York: Routledge.

[ref39] Konradt U. Otte K.-P. Schippers M. C. Steenfatt C. (2016). Reflexivity in teams: a review and new perspectives. J. Psychol. 150, 153–174. doi: 10.1080/00223980.2015.1050977, PMID: 26457836

[ref40] Konradt U. Schippers M. C. Garbers Y. Steenfatt C. (2015). Effects of guided reflexivity and team feedback on team performance improvement: the role of team regulatory processes and cognitive emergent states. Eur. J. Work Organ. Psy. 24, 777–795. doi: 10.1080/1359432X.2015.1005608

[ref45] Kramer R. Tyler T. Lewicki R. Bunker B. (1996). In Developing and maintaining trust in work relationships. SAGE Publications, Inc., 114–139. doi: 10.4135/9781452243610

[ref41] Krull J. L. MacKinnon D. P. (2001). Multilevel modeling of individual and group level mediated effects. Multivar. Behav. Res. 36, 249–277. doi: 10.1207/S15327906MBR3602_06, PMID: 26822111

[ref42] Lambooij M. Flache A. Sanders K. Siegers J. (2007). Encouraging employees to co-operate: the effects of sponsored training and promotion practices on employees' willingness to work overtime. Int. J. Hum. Resour. Manag. 18, 1748–1767. doi: 10.1080/09585190701570932

[ref43] Leblanc P. M. Rousseau V. Harvey J. F. (2022). Leader humility and team innovation: the role of team reflexivity and team proactive personality. J. Organ. Behav. 43, 1396–1409. doi: 10.1002/job.2648

[ref44] LeBreton J. M. Senter J. L. (2008). Answers to 20 questions about interrater reliability and interrater agreement. Organ. Res. Methods 11, 815–852. doi: 10.1177/1094428106296642

[ref46] Liang H.-Y. Shih H.-A. Chiang Y.-H. (2015). Team diversity and team helping behavior: the mediating roles of team cooperation and team cohesion. Eur. Manag. J. 33, 48–59. doi: 10.1016/j.emj.2014.07.002

[ref47] Lin C.-P. Joe S.-W. Tsai Y. H. Huang C.-C. (2013). Exploring team climate and performance: mediating effects of cooperation and team efficacy. ASBBS Proc. 20:309.

[ref48] Lin M. Zhang X. Ng B. C. S. Zhong L. (2022). The dual influences of team cooperative and competitive orientations on the relationship between empowering leadership and team innovative behaviors. Int. J. Hosp. Manag. 102:103160. doi: 10.1016/j.ijhm.2022.103160

[ref49] Marks M. A. Mathieu J. E. Zaccaro S. J. (2001). A temporally based framework and taxonomy of team processes. Acad. Manag. Rev. 26, 356–376. doi: 10.2307/259182

[ref50] Mayer R. C. Davis J. H. Schoorman F. D. (1995). An integrative model of organizational trust. Acad. Manag. Rev. 20, 709–734. doi: 10.2307/258792

[ref51] Maynard M. T. Kennedy D. M. Resick C. J. (2018). Teamwork in extreme environments: lessons, challenges, and opportunities. J. Organ. Behav. 39, 695–700. doi: 10.1002/job.2302

[ref52] Mohammed S. Ferzandi L. Hamilton K. (2010). Metaphor no more: a 15-year review of the team mental model construct. J. Manag. 36, 876–910. doi: 10.1177/0149206309356804

[ref53] Mohrman S. A. Lawler E. E. (1989). Parallel participation structures. Public Adm. Q. 13, 255–272.

[ref54] Mulyani S. R. Sari V. N. Sari M. W. (2020). Model of employee motivation and cooperative performance. Utopía y Praxis Latinoamericana 25, 232–242. doi: 10.5281/zenodo.3774631

[ref55] Ng E. S. W. Schweitzer L. Lyons S. T. (2010). New generation, great expectations: a field study of the millennial generation. J. Bus. Psychol. 25, 281–292. doi: 10.1007/s10869-010-9159-4

[ref56] Nunnally J. Bernstein I. (1994). Psychometric theory. 3rd Edn. New York: McGraw-Hill.

[ref57] Nyhan R. C. Marlowe H. A. Jr. (1997). Development and psychometric properties of the organizational trust inventory. Eval. Rev. 21, 614–635. doi: 10.1177/0193841X9702100505

[ref58] Ostroff C. Bowen D.E. (2000). Moving HR to a higher level: HR practices and organizational effectiveness. In: Multilevel theory, research, and methods in organizations: Foundations, extensions, and new directions. eds. Klein K. J. Kozlowski S. W. J. eds. Jossey-Bass/Wiley. 211–266.

[ref59] Özer Ö. Zheng Y. Chen K.-Y. (2011). Trust in forecast information sharing. Manag. Sci. 57, 1111–1137. doi: 10.1287/mnsc.1110.1334, PMID: 19642375

[ref60] Parker S. K. Mullarkey S. Jackson P. (1994). Dimensions of performance effectiveness in high-involvement work organisations. Hum. Resour. Manag. J. 4, 1–21. doi: 10.1111/j.1748-8583.1994.tb00342.x

[ref61] Peñarroja V. Orengo V. Zornoza A. Hernández A. (2013). The effects of virtuality level on task-related collaborative behaviors: the mediating role of team trust. Comput. Hum. Behav. 29, 967–974. doi: 10.1016/j.chb.2012.12.020

[ref62] Preacher K. J. Rucker D. D. Hayes A. F. (2007). Addressing moderated mediation hypotheses: theory, methods, and prescriptions. Multivar. Behav. Res. 42, 185–227. doi: 10.1080/00273170701341316, PMID: 26821081

[ref63] Rawlins B. (2008). Measuring the relationship between organizational transparency and employee trust. Public Relat. J. 2, 1–21.

[ref64] Ren S. Xie Y. Zhu Y. Warner M. (2018). New generation employees’ preferences towards leadership style in China. Asia Pac. Bus. Rev. 24, 437–458. doi: 10.1080/13602381.2018.1451128

[ref65] Riordan C. M. Vandenberg R. J. Richardson H. A. (2005). Employee involvement climate and organizational effectiveness. Hum. Resour. Manag. 44, 471–488. doi: 10.1002/hrm.20085

[ref66] Romano A. Balliet D. (2017). Reciprocity outperforms conformity to promote cooperation. Psychol. Sci. 28, 1490–1502. doi: 10.1177/0956797617714828, PMID: 28877004

[ref67] Salancik G. R. Pfeffer J. (1978). A social information processing approach to job attitudes and task design. Adm. Sci. Q. 23, 224–253. doi: 10.2307/2392563 10307892

[ref68] Salas E. Shuffler M. L. Thayer A. L. Bedwell W. L. Lazzara E. H. (2015). Understanding and improving teamwork in organizations: a scientifically based practical guide. Hum. Resour. Manag. 54, 599–622. doi: 10.1002/hrm.21628

[ref69] Santos S. C. Neumeyer X. (2022). Culture and gender in entrepreneurial teams: the effect on team processes and outcomes. Small Bus. Econ. 58, 1035–1050. doi: 10.1007/s11187-020-00432-x

[ref70] Schippers M. C. Den Hartog D. N. Koopman P. L. Wienk J. A. (2003). Diversity and team outcomes: the moderating effects of outcome interdependence and group longevity and the mediating effect of reflexivity. J. Org. Behav. 24, 779–802. doi: 10.1002/job.220

[ref71] Schippers M. C. West M. A. Dawson J. F. (2015). Team reflexivity and innovation: the moderating role of team context. J. Manag. 41, 769–788. doi: 10.1177/0149206312441210

[ref72] Simmons C. H. Wehner E. A. Tucker S. S. King C. S. (1988). The cooperative/competitive strategy scale: a measure of motivation to use cooperative or competitive strategies for success. J. Soc. Psychol. 128, 199–205. doi: 10.1080/00224545.1988.9711363

[ref73] Simon H. A. (1978). “Information-processing theory of human problem solving” in Handbook of learning and cognitive processes, W. K. Estes (Ed.). New York, Hilsdale: Erlbaum. vol. 5, 271–295.

[ref74] Smith-Jentsch K. A. Cannon-Bowers J. A. Tannenbaum S. I. Salas E. (2008). Guided team self-correction: impacts on team mental models, processes, and effectiveness. Small Group Res. 39, 303–327. doi: 10.1177/1046496408317794

[ref75] Stamper C. L. Masterson S. S. (2002). Insider or outsider? How employee perceptions of insider status affect their work behavior. J. Org. Behav. 23, 875–894. doi: 10.1002/job.175

[ref76] Swift T. A. West M. A. (1998). Reflexivity and group processes: research and practice. Sheffield: ESRC Centre for Organization and Innovation.

[ref77] Tannenbaum S. I. Cerasoli C. P. (2013). Do team and individual debriefs enhance performance? A meta-analysis. Hum. Factors 55, 231–245. doi: 10.1177/0018720812448394, PMID: 23516804

[ref78] Teece D. J. Pisano G. Shuen A. (1997). Dynamic capabilities and strategic management. Strateg. Manag. J. 18, 509–533. doi: 10.1002/(SICI)1097-0266(199708)18:7<509::AID-SMJ882>3.0.CO;2-Z

[ref79] Van den Bossche P. Gijselaers W. Segers M. Woltjer G. Kirschner P. (2011). Team learning: building shared mental models. Instr. Sci. 39, 283–301. doi: 10.1007/s11251-010-9128-3

[ref80] van Gerwen N. Buskens V. van der Lippe T. (2018a). Employee cooperative behavior in organizations: a vignette experiment on the relationship between training and helping intentions. Int. J. Train. Dev. 22, 192–209. doi: 10.1111/ijtd.12128, PMID: 31543696 PMC6743710

[ref81] van Gerwen N. Buskens V. van der Lippe T. (2018b). Individual training and employees’ cooperative behavior: evidence from a contextualized laboratory experiment. Ration. Soc. 30, 432–462. doi: 10.1177/1043463118771428

[ref82] Vashdi D. R. Bamberger P. A. Erez M. Weiss-Meilik A. (2007). Briefing-debriefing: using a reflexive organizational learning model from the military to enhance the performance of surgical teams. Hum. Resour. Manag. 46, 115–142. doi: 10.1002/hrm.20148

[ref83] Wang Z. Ren S. Chadee D. Liu M. Cai S. (2021). Team reflexivity and employee innovative behavior: the mediating role of knowledge sharing and moderating role of leadership. J. Knowl. Manag. 25, 1619–1639. doi: 10.1108/JKM-09-2020-0683

[ref84] West M. (1996). Reflexivity and work group effectiveness: a conceptual integration. Chichester: John Wiley & Sons, Ltd.

[ref85] West M. Garrod S. Carletta J. (1997). Group decision-making and effectiveness: unexplored boundaries. in Creating tomorrow’s organizations: a handbook for future research in organizational behavior. eds. Cooper C. L. Jackson S. E.. Chichester: John Wiley & Sons, Ltd,. 293–317.

[ref86] Yang M. Schloemer H. Zhu Z. Lin Y. Chen W. Dong N. (2020). Why and when team reflexivity contributes to team performance: a moderated mediation model. Front. Psychol. 10:3044. doi: 10.3389/fpsyg.2019.03044, PMID: 32038407 PMC6985579

[ref87] Ye S. Xiao Y. Yang B. Zhang D. (2021). The impact mechanism of entrepreneurial team expertise heterogeneity on entrepreneurial decision. Front. Psychol. 12:732857. doi: 10.3389/fpsyg.2021.732857, PMID: 34671301 PMC8521117

[ref88] Zhu Y. Xie Y. Warner M. Guo Y. (2015). Employee participation and the influence on job satisfaction of the ‘new generation’ of Chinese employees. Int. J. Hum. Resour. Manag. 26, 2395–2411. doi: 10.1080/09585192.2014.990397

